# Ninth Quarterly Meeting of the Austin District Medical Association

**Published:** 1890-01

**Authors:** T. J. Bennett

**Affiliations:** Secretary; Austin


### Society Notes.

### THE NINTH QUARTERLY MEETING OF THE AUSTIN DISTRICT MEDICAL SOCIETY.

AUSTIN, DECEMBER 19, 1889.

Dr. W. A. Morris, the retiring President called the Society to order at 9:30 a. m., and introduced the President-elect, Dr. F. R. Martin, of Kyle.

Dr. Martin made some very appropriate remarks. He thanked the society for the unexpected compliment, and promised to discharge the duty of President to the best of his ability.

The following names were added to the membership :

Dr. A. Garwood, of New Braunfels.

Dr. W. V. Hickman, Smithville.

Dr. A. J. Culbertson, Burnet.

Dr. J. F. Wilson, Bertram.

Dr. J. W. Carhart, Lampasas.

Dr. H. B. Hill, Austin.

Dr. J. F. Perry, Red Rock.

Dr. R. Steiner, Austin.

Dr. W. B. Gibson, Austin.

Dr. W. A. Morris read his retiring address entitled: "Man."* It was regarded as one of the ablest addresses ever made before this body. It dealt with man's capabilities, and his advancement in a scientific point since the author entered upon his professional career fifty-eight years ago.

An old man can tell us younger men a great many things that will interest us. Dr. Morris has shown by his address that he has been a close student, and has kept up with the times in every department of scientific research.

Dr. C. O. Weller read a paper entitled: "Phlegmasia Dolens." It was discussed by Drs. W. M. Cunningham, J. W. McLaughlin, R. S. Gregg, A. J. Davis, M. A. Taylor, J. W. Carhart, G. W. Christian and W. A. Morris.†

Dr. J. W. Carhart read a report of a case of complete closure of both external auditory canals by bony structure. Dr. Way made remarks upon the paper.

Dr. J. W. McLaughlin read a paper on "Salpingitis," which was generally discussed, especially by Drs. Christian, Taylor and Carhart.

The next paper was read by Dr. G. B. Underhill, entitled: "Continued Fever." An interesting discussion followed, par-

*The address will appear in the March number of the JOURNAL.

†The discussions on all the papers will appear following the papers as they are published.

ticipated in by Drs. Morris, McLaughlin, Christian, Taylor, Tyner and Wooten.

Dr. Joe Cummings read a paper on "Tonsilitis." Remarks were made upon the paper by Drs. W. H. Way, Tyner, Morris and Wooten.

Dr. Christian read a letter from a citizen of Burnet county, describing a new "water filter." Dr. Christian was asked by the society to explain the instrument, which he did, by saying that it was proposed to purify the water, as it was pumped up, by the use of a volume of air through which the water was forced, on the same principle that water in motion, as in a river, is purified by the air it comes in contact with as it flows.

On motion of Dr. Wooten the "patent" was referred to the "State Board of Health."

On motion Drs. Swearingen, Morris and McLaughlin were appointed a committee to confer with Dr. T. D. Wooten, President of the Board of Regents of the State University relative to the medical department. The committee to report at the March meeting of the Society.

Dr. Bennett reported as chairman of a committee from Travis County Medical Society on the subject of permanent quarters for the two societies. It was estimated that a certain hall on Congress Avenue, 56x21 feet, would cost $125 per annum, and that it would cost about $300 to properly furnish it so the societies could occupy it. Cabinets for pathological specimens and additional library facilities would be extra. A committee was appointed, after the Society had discussed and endorsed the movement, to enter into agreement with Travis County Medical Society, and to select and properly furnish a suitable hall by the next meeting of the Society. Committee: Drs. Bennett, Swearingen, Morris and Wooten.

Each member was assessed three dollars for the above purpose, and requested to hand same at once to the Secretary.

A banquet was served at night.

T. J. BENNETT, Secretary.

